# Kinase domain activation through gene rearrangement in multiple myeloma

**DOI:** 10.1038/s41375-018-0108-y

**Published:** 2018-03-23

**Authors:** Gareth J Morgan, Jie He, Ruslana Tytarenko, Purvi Patel, Owen W Stephens, Shan Zhong, Shayu Deshpande, Michael Bauer, Niels Weinhold, Carolina Schinke, Leo Rasche, Mark Bailey, Siraj Ali, Jeff Ross, Vincent A Miller, Phillip Stephens, Sharmilan Thanendrarajan, Maurizio Zangari, Frits van Rhee, Tariq Mughal, Faith E Davies, Brian A Walker

**Affiliations:** 1 The Myeloma Institute, University of Arkansas for Medical Sciences, Little Rock, AR USA; 2 Foundation Medicine Inc., Cambridge, MA USA; 3 Albany Medical College, Albany, NY USA; 4 Tufts University Medical Center, Boston, MA USA

## Abstract

Chromosomal rearrangements that result in oncogenic kinase activation are present in many solid and hematological malignancies, but none have been reported in multiple myeloma (MM). Here we analyzed 1421 samples from 958 myeloma patients using a targeted assay and detected fusion genes in 1.5% of patients. These fusion genes were in-frame and the majority of them contained kinase domains from either receptor tyrosine kinases (*ALK*, *ROS1*, *NTRK3*, and *FGFR1*) or cytoplasmic kinases (*BRAF*,* MAP3K14*, and *MAPK14*), which would result in the activation of MEK/ERK, NF-κB, or inflammatory signaling pathways. Fusion genes were present in smoldering MM, newly diagnosed MM, and relapse patient samples indicating they are not solely late events. Most fusion genes were subclonal in nature, but one *EML4-ALK* fusion was clonal indicating it is a driver of disease pathogenesis. Samples with fusions of receptor tyrosine kinases were not found in conjunction with clonal Ras/Raf mutations indicating a parallel mechanism of MEK/ERK pathway activation. Fusion genes involving *MAP3K14* (*NIK*), which regulates the NF-κB pathway, were detected as were t(14;17) rearrangements involving *NIK* in 2% of MM samples. Activation of kinases in myeloma through rearrangements presents an opportunity to use treatments existing in other cancers.

## Introduction

Fusion genes are the product of genomic rearrangements where two genes are rearranged to create a new fusion gene with either increased or inappropriate expression and functionality that was not previously evident. One of the initial rearrangements defining fusion genes was the t(9;22), typical of chronic myeloid leukemia (CML) cases [1, 2]. This translocation generates a *BCR-ABL1* fusion gene, which results in the activation of the tyrosine kinase domain of the ABL protein. The increased signaling, which occurs as a result of this rearrangement, can be therapeutically targeted by specific tyrosine kinase inhibitors (TKIs), resulting in clinically relevant responses [3, 4].

In solid tumors, fusion genes are also seen and similarly can result in the activation of kinase domains with key deregulated genes including the receptor tyrosine kinases (RTKs) *ALK*, *ROS1*, *RET*, *FGFR1/2/3*, and *NTRK1/2/3*, as well as cytoplasmic kinases such as *BRAF* [5, 6]. These rearrangements are not as frequent as the t(9;22) with rates of up to 12% in patients with thyroid carcinoma, but on the whole, across all types of cancer, the rate is in the order of 1–2% [7]. The most common signaling pathway deregulated by these fusion genes is the MEK/ERK pathway. Analogous to CML, these kinase fusion genes can also be therapeutically targeted, a key example of which is the *EML4-ALK* fusion gene seen in 3–7% of patients with adenocarcinoma of the lung [8].

Multiple myeloma (MM) is characterized by primary translocations into the immunoglobulin (Ig) loci, which occur in ~40% of patients [9]. These structural rearrangements place the Ig super-enhancer next to an oncogene, resulting in its overexpression [10]. The common gene fusions seen in MM involve the Ig loci, including cases with a t(4;14) where the breakpoint within *MMSET* results in an *Ig-MMSET* transcript, but an alteration in functionality as a consequence is not seen [11, 12]. A key signaling abnormality in MM is increased MEK/ERK pathway activation due to activating point mutations in *KRAS*, *NRAS*, and *BRAF* seen in up to 50% of patients [13]. However, despite the frequent deregulation of this pathway, functional gene fusions involving RTKs have not been described. Here we analyzed 1421 patient samples using a targeted assay able to detect gene fusions and describe their prevalence in MM.

## Methods

### Patient samples and nucleic acid extraction

We report on 1421 samples from 958 individuals diagnosed with monoclonal gammopathy of undetermined significance (MGUS), smoldering multiple myeloma (SMM), or MM who underwent targeted sequencing with the FoundationOne heme (F1H) assay [14, 15] between September 2013 and August 2016. All patients signed a written informed consent in keeping with institutional, federal, and Helsinki Declaration guidelines. Tumor samples were obtained from bone marrow aspirates, enriched by CD138^+^ selection using magnetic beads (AutoMACs, Miltenyi Biotech, Cologne, Germany or RoboSep, StemCell Technologies, Vancouver, Canada). RNA and DNA were extracted using the AllPrep DNA/RNA mini kit (Qiagen, Hilden, Germany), RNeasy RNA extraction kit (Qiagen), or Puregene DNA extraction kit (Qiagen).

### Foundation One heme reporting

DNA ≥50 ng and/or RNA >300 ng was interrogated using the F1H Panel (Foundation Medicine, MA). The panel analyzes the complete coding DNA sequence of 405 genes, as well as selected introns of 31 genes involved in chromosomal rearrangements. It also interrogates the RNA sequence of 265 commonly rearranged genes resulting in gene fusions. Genes included in this assay encode known or likely targets of therapy, either FDA-approved or in clinical trials, or are otherwise known drivers of oncogenesis. Due to the capture strategies, most fusions are detected by either DNA or RNA sequencing and not both. Of the 1421 samples, 565 were processed on the DNA panel only and 856 were processed on the DNA and RNA panels. Data have been submitted to the European Genome-Phenome Archive  under accession EGAS00001002874.

Sequencing was carried out to an average depth of 468x and was performed using a HiSeq 2500 (Illumina). Sequences were analyzed for base substitutions, indels, copy-number alterations (focal amplifications with ≥8 copies and homozygous deletions), and selected gene rearrangements. Variant processing is described elsewhere [14, 16], but importantly involved removal of germ line variants from the 1000 Genomes Project (dbSNP135), as a matched patient non-tumor sample is not used to identify truly somatic variants. All inactivating events (i.e., truncations and deletions) in known tumor suppressor genes were also called as significant. To maximize mutation-detection accuracy (sensitivity and specificity) in clinical specimens, the test has been optimized and validated to detect base substitutions at a ≥5% variant allele frequency (VAF) and indels with a ≥10% VAF to ≥99% accuracy. However, mutations are reported down to 1% VAF where the variant is a known hotspot and there is sufficient purity and sequencing depth. Reports were generated by Foundation Medicine and data files containing additional information (VAF, variant type, depth at variant location, genomic coordinates) were received.

### RT-PCR confirmation of fusion genes

To confirm the fusion gene breakpoints, reverse transcription-polymerase chain reaction (RT-PCR) was performed on seven samples. Complementary DNA (cDNA) was synthesized from 200 ng RNA using iScript (Bio-Rad). Two microliters of a 1/10 dilution of the cDNA was used in subsequent PCR reactions. PCR bands were excised from agarose gels, purified, and sequenced by the Sanger method. Sanger sequence reads were compared against the genome using BLAST.

### Determining clonality of fusion genes

Custom probe-based qPCR assays (Integrated DNA Technologies) were designed across the DNA breakpoints in order to determine if the fusion genes are clonal. Assays were also designed against the unarranged germ line sequence for comparison, as well as for any Ig translocations that had also been detected in the same samples. Only samples with an identified DNA breakpoint were analyzed as the sequence is required to design the assays. Rearranged and germ line sequence assays were labeled with FAM or HEX, respectively. DNA from the samples was used in a digital droplet PCR reaction to determine the proportion of sample with a rearrangement. If a primary IGH translocation breakpoint was assayed, it was assumed to be clonal and the percent positive droplets in the IGH and fusion gene assay adjusted to account for CD138 cell purity.

### Gene expression profiling

Gene expression profiling (GEP) using Affymetrix U133 Plus 2.0 arrays was performed. The GEP-based 70 risk score (GEP70) and molecular subgroups were determined as previously described [17, 18]. Dataset GSE4581 was used to determine overexpression of *MAP3K14*. Overexpression was determined as values with >2 standard deviations of the mean for probeset 205192_at.

### CoMMpass dataset validation

Data are available at dbGap under accession number phs000748.v5.p4. RNA-seq data were processed by STAR and Salmon. Gene level quantification of RNA expression was performed using Star (2.5.1b) generating read counts per gene while aligning the reads to the reference genome (hg38). Salmon (0.6.0) was run to calculate transcript level quantification. Additionally, the Salmon transcript values were summed to get quantification at the gene level.

Previously, aligned BAM files from whole-genome sequencing (WGS) were converted to FASTQ using Picard tools v2.1.1 to extract read sequences and base quality scores. All reads were realigned to the human genome assembly hg19 using BWA-mem. Base recalibration of alignments was performed using GATK v3.6. Translocations in whole-genome data were detected using Manta (version 0.29.6) with default settings.

Using the CoMMpass dataset, comprising 564 RNA-seq samples that have matching WGS, fusion genes were identified using MapSplice 2 (v2.2.1). The results were parsed to identify relevant kinase fusion genes. The identified fusion genes were confirmed using the WGS data where the translocation breakpoints at the DNA level were identified using Manta (v0.29.6).

## Results

### Identification of fusion genes in multiple myeloma

Using a capture panel for both RNA and DNA, 1421 samples from 958 patients were sequenced for clinical purposes. From these data, 39 potential fusion genes were identified and annotated from either their DNA breakpoints (*n* = 11) or RNA sequences matching multiple genes (*n* = 31) or both (*n* = 3). Of the 39 potential fusion genes, 21 were in-frame, potentially resulting in functional fusion proteins (Table 1). The patients with in-frame fusions included those with SMM (*n* = 1), newly diagnosed MM (*n* = 5), those who had been previously treated (*n* = 2), and those who had relapsed (*n* = 11).

**Table 1:** In-frame fusion genes identified by RNA or DNA sequencing

Sample	Disease state	Cytogenetic group	GEP70 risk group	Head gene	Head last exon	Tail gene	Tail first exon	In-frame	RNA-seq	RT-PCR	DNA-seq	ddPCR	Fusion gene function
35882	SMM	t(4;14)	LR	*TSPAN3*	5	*ROS1*	31	Yes	Yes	Yes	NC	ND	Kinase
38203	NDMM	HRD	LR	*ATM*	57	*DLG2*	8	Yes	No	ND	Yes	ND	Kinase
35830	NDMM	HRD	LR	*TXNDC5*	7	*MYC*	2	Yes	Yes	ND	No	ND	TF
37871	NDMM	t(14;20)	LR	*KANK1*	7	*BRAF*	9	Yes	No	ND	Yes	Yes	Kinase
39882	NDMM	t(4;14)	LR	*SUB1*	3	*WHSC1*	5	Yes	Yes	ND	NC	ND	
40145	NDMM	HRD	LR	*MED15*	1	*EP300*	21	Yes	ND	ND	Yes	ND	TF
35711	Treated	t(14;20)	HR	*CDC6*	9	*RARA*	3	Yes	No	ND	Yes	ND	TF
38740	Treated	t(14;16)	HR	*EML4*	6	*ALK*	20	Yes	Yes	Yes	Yes	Yes	Kinase
15639	Relapse	HRD	LR	*SS18*	5	*FLI1*	4	Yes	Yes	ND	NC	ND	TF
10763	Relapse	t(4;14)	LR	*GTF2I*	4	*BRAF*	10	Yes	Yes	ND	Yes	Yes	Kinase
10763	Relapse	t(4;14)	LR	*AGK*	2	*BRAF*	8	Yes	Yes	Yes	No	ND	Kinase
10763	Relapse	t(4;14)	LR	*SNX29*	1	*FGFR1*	4	Yes	Yes	ND	NC	ND	Kinase
14122	Relapse	HRD	LR	*HNRNPA2B1*	7	*NTRK3*	14	Yes	Yes	Yes	Yes	ND	Kinase
21003	Relapse	t(4;14)	HR	*SUB1*	3	*WHSC1*	5	Yes	Yes	ND	NC	ND	
21058	Relapse	t(11;14)	LR	*AKT1*	2	*MAPK14*	11	Yes	Yes	Yes	NC	ND	Kinase
23130	Relapse	HRD	LR	*FOXO3*	1	*MYC*	2	Yes	Yes	ND	Yes	ND	TF
27259	Relapse	t(4;14)	HR	*ESYT2*	8	*BRAF*	9	Yes	Yes	Yes	No	ND	Kinase
38731	Relapse	t(11;14)	HR	*SLC5A5*	7	*MYO18A*	8	Yes	NC	ND	Yes	ND	
39240	Relapse	HRD	HR	*TXNDC5*	5	*MYC*	2	Yes	Yes	ND	Yes	ND	TF
39451	Relapse	t(14;16)	HR	*UBE2R2*	3	*NTRK3*	4	Yes	Yes	Yes	NC	ND	Kinase
15933	Relapse	HRD	LR	*ARHGAP27*	6	*MAP3K14*	5	Yes	NC	ND	Yes	Yes	Kinase

*ND* assay not performed, *NC* assay performed but not captured on panel, *SMM* smoldering multiple myeloma, *LR* low risk, *HR* high risk, *HRD* hyperdiploid, *TF* transcription factor

### Classes of fusion genes

The panel detects known common kinase and transcription factor fusions, and of the 21 in-frame fusion genes, 12 involved kinases, 6 involved transcription factors, and 3 were neither. Of the 3 that were neither kinases or transcription factors, 2 involved *MMSET/WHSC1* and were both in t(4;14) patient samples. Of the 6 involving transcription factors, 3 involved *MYC* with common partners of *MYC* rearrangements in MM (*TXNDC5* and *FOXO3*) and were seen in the hyperdiploid samples.

**Fig. 1:**
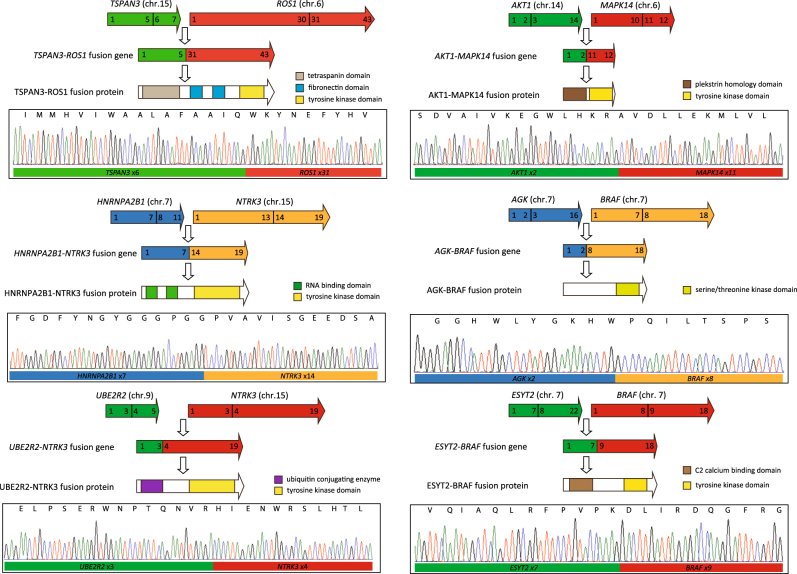
Confirmation of fusion gene breakpoints. RNA from samples with the selected fusion gene underwent RT-PCR followed by Sanger sequencing. Cartoons of the expected fusion rearrangements and the electropherograms confirming the breakpoint are shown for six samples

**Fig. 2:**
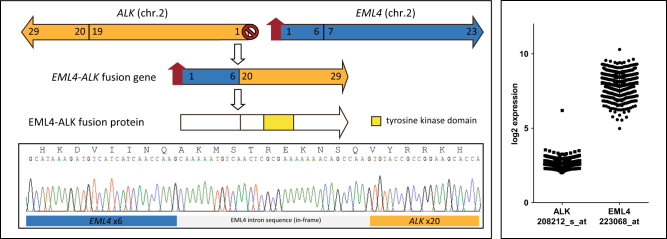
*EML4-ALK* rearrangement in myeloma results in increased expression of the tyrosine kinase domain. *EML4* and *ALK* are in opposite transcriptional orientation on chromosome 2. An inversion results in the active promoter from *EML4* being placed upstream of the 5′ end of *ALK*, resulting in expression of the *EML4-ALK* fusion protein containing a tyrosine kinase domain. The fusion gene was confirmed by RT-PCR and Sanger sequencing. *ALK* is not expressed in adult tissue but *EML4* is, however, in this sample, expression of the *EML4-ALK* fusion transcript was detected using 3′ expression array analysis

Where a kinase domain was involved, it was located at the 3′ end of the fusion gene, which is common for this type of fusion. The most common partner identified was *BRAF* (*n* = 4), followed by *NTRK3* (*n* = 2). All other kinase fusion genes were unique and included *ALK*, *FGFR1*, *MAP3K14*, *MAPK14*, and *ROS1*. As expected, all fusions retained the kinase domain. Seven samples had RNA available and the fusion genes were verified by RT-PCR (Figs. 1 and 2). Sanger sequencing identified the breakpoints in the messenger RNA and schematics of the fusion genes are shown.

The kinases involved in the fusion genes have been noted in other cancers, including *BRAF* (papillary thyroid cancer, cutaneous melanoma, and adenocarcinoma of the rectum), *NTRK3* (thyroid, melanoma, colon adenocarcinoma, and invasive breast cancer), *FGFR1* (adenocarcinoma of the lung and breast cancer), *MAP3K14* (head and neck squamous cell carcinoma and ovarian cancer), *ROS1* and *ALK* (adenocarcinoma of the lung).

*ALK* fusion genes in lung adenocarcinoma involve an inversion on chromosome 2, placing the promoter of *EML4* in front of *ALK*. *ALK* is not normally expressed in adult tissue, whereas *EML4* is expressed. The inversion of the promoters results in the *EML4-ALK* fusion gene and overexpression of the kinase domain within *ALK*. As we found an *EML4*-*ALK* fusion in our dataset, we used GEP data to determine if *ALK* was overexpressed. As expected, *ALK* was not expressed in any sample except for the one with the inversion (Fig. 2).

The kinase fusions fall into two categories: RTKs and cytoplasmic kinases. ROS1, ALK, FGFR1, and NTRK3 are all RTKs that are known to result in downstream MEK/ERK or PIK3CA signaling pathway activation leading to cell proliferation or prevent apoptosis. The cytoplasmic kinases are more diverse, with BRAF also being a member of the MEK/ERK activation pathway, whereas MAPK14 (p38) and MAP3K14, also known as NF-κB-inducing kinase (NIK), are involved in the inflammatory and NF-κB signaling pathways, respectively.

**Fig. 3:**
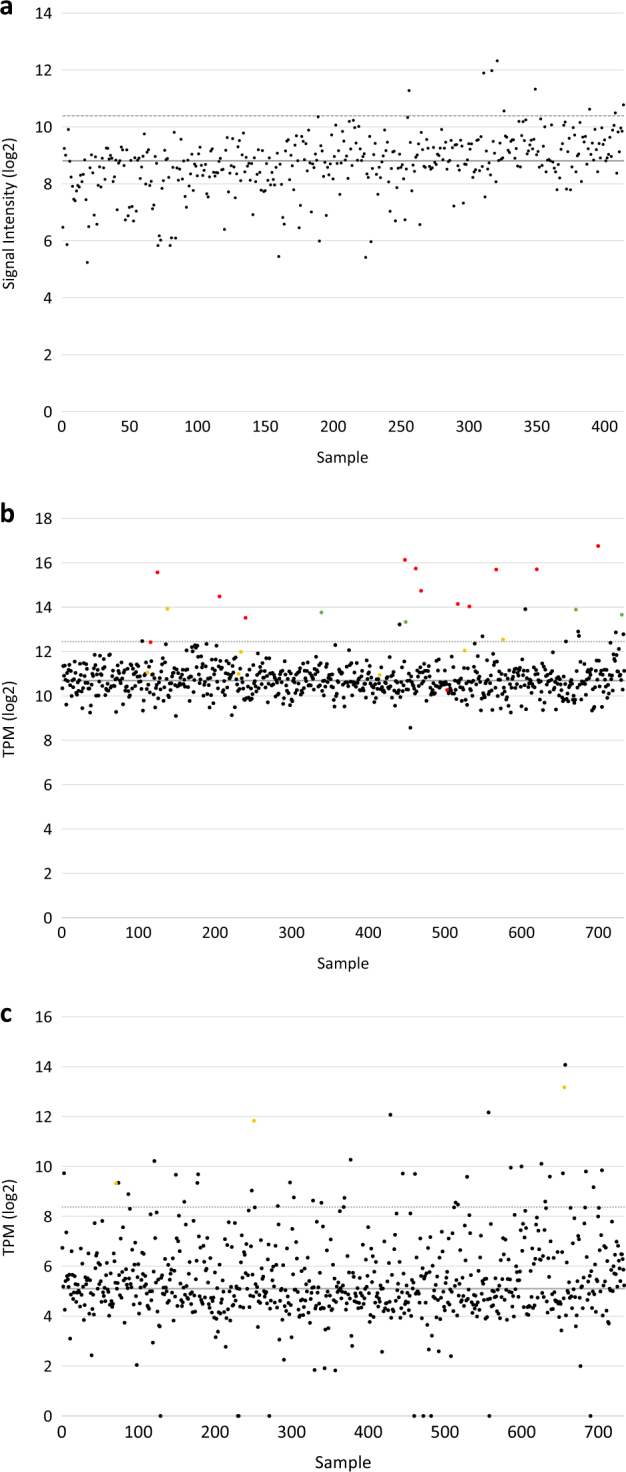
Expression of *MAP3K14* and *NTRK1* in newly diagnosed myeloma patient samples. **a** Spiked expression of probeset 205192_at on U133 Plus 2.0 arrays was used as a surrogate for the t(14;17). **b** Spiked expression of *MAP3K14* in the CoMMpass RNA-seq dataset. **c** Spiked expression of *NTRK1* in the CoMMpass RNA-seq dataset. Red and green circles indicate presence of an Ig or non-Ig translocation in the WGS data, respectively. Yellow circles are samples with a *MAP3K14* or *NTRK1* fusion gene in RNA-seq data. The solid gray line indicates the median expression of the probeset across all samples. The dotted gray line indicates the median plus 2 standard deviations and was used as the cutoff to determine spiked expression

We analyzed the CoMMpass dataset comprising 487 patients and identified a similar spectrum of kinase fusion genes with evidence both in RNA and DNA sequencing including *SND1-BRAF*,* TPR-NTRK1*,* FCHSD2-MAP3K14*,* TPM3-NTRK1*,* CREB1-ALK*,* IKZF3-MAP3K14*, *ARHGEF2-NTRK1*, and *BRAF-AGK* (Table 2). These were present in a total of 2.5% of patients. Expression of the kinase gene was not always increased as a result of the fusion, e.g., in *BRAF* and *MAP3K14* fusions (Fig. 3a, b), but was in others (*NTRK1* (0.5% of samples) and *ALK*; Figs. 2 and 3c). The most frequent kinase fusion domain in this dataset involved NIK, where the N-terminus of NIK is replaced by different head genes. This may result in stabilization of NIK through loss of the N-terminal BIRC2 (cIAP-1)-binding domain that regulates degradation of NIK [19].

**Table 2:** Kinase fusion genes identified in the CoMMpass dataset

Sample	Cytogenetic group	IMWG risk	Head gene	Head last exon	Tail gene	Tail first exon	In-frame
MMRF_1032_1	HRD	Standard	*SND1*	10	*BRAF*	11	Yes
MMRF_1232_4	HRD	ND	*TPR*	10	*NTRK1*	3	Yes
MMRF_1331_1	HRD	Standard	*CDC27*	4	*MAP3K14*	4	Yes
MMRF_1392_1	t(4;14)	High	*FCHSD2*	2	*MAP3K14*	4	Yes
MMRF_1618_1	t(14;16)	ND	*NMT1*	1	*MAP3K14*	4	Yes
MMRF_1625_1	t(14;20)	Standard	*EFTUD2*	11	*MAP3K14*	3	Yes
MMRF_1656_1	HRD	Standard	*TPM3*	6	*NTRK1*	10	Yes
MMRF_1846_1	t(11;14)	Standard	*CREB1*	1	*ALK*	9	Yes
MMRF_2000_1	t(11;14)	Low	*TAF15*	3	*MAP3K14*	6	Yes
MMRF_2272_1	t(4;14)	High	*YBX1*	2	*MAP3K14*	4	Yes
MMRF_2412_1	HRD	Standard	*IKZF3*	1	*MAP3K14*	4	Yes
MMRF_2490_1	t(12;14)	Standard	*ARHGEF2*	21	*NTRK1*	12	Yes
MMRF_1783_2	t(4;14)	High	*BRAF*	7	*AGK*	3	Yes

*ND* not determined

### Association of kinase fusion genes with other MEK/ERK pathway abnormalities

In myeloma, recurrent mutations affect Ras signaling leading to activation of the MEK/ERK pathway. *KRAS*, *NRAS*, and *BRAF* are most frequently mutated and collectively are present in up to 50% of patients. It is known that mutations in these three genes are mutually exclusive, although subclones with different mutations within a patient do exist. Using the data from the F1H assay, we determined if samples also had concomitant kinase fusions and MEK/ERK pathway mutations (Table 3). Five samples had both a kinase fusion and mutation of *Ras*/*Raf* genes, however, all but one were at low VAFs indicating they were subclonal.

**Table 3:** Concurrent Ras mutations and kinase fusion genes

Fusion	Ras/Raf mutation (variant allele frequency)	IGH translocation	GEP70 risk
*AGK-BRAF* ^a^	None	t(4;14)	Low
*AKT1-MAPK14*	None	t(11;14)	Low
*ARHGAP27-MAP3K14*	*KRAS* (2%), *NRAS* (2%)	t(4;14)	High
*EML4-ALK*	None	t(14;16)	High
*ESYT2-BRAF*	None	t(4;14)	High
*GTF2I-BRAF* ^a^	*KRAS* (16%)	t(4;14)	Low
*HNRNPA2B1-NTRK3*	None	None	Low
*KANK1-BRAF*	*BRAF* (2%)	t(14;20)	Low
*SNX29-FGFR1* ^a^	None	t(4;14)	Low
*TSPAN3-ROS1*	*BRAF* (2%)	t(4;14)	High
*UBE2R2-NTRK3*	*KRAS* (42%)	t(14;16)	High
*ATM-DLG2*	None	None	Low

^a^ Same patient at different time points

**Fig. 4:**
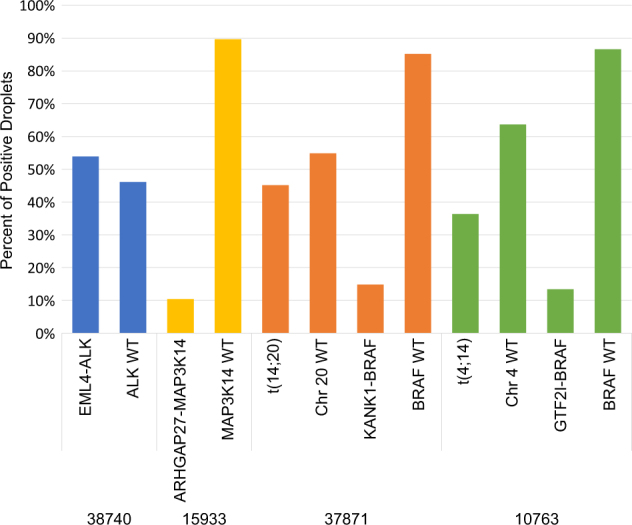
Kinase gene fusions can be both clonal or subclonal. Clonality of fusion gene breakpoints was determined using droplet digital PCR (ddPCR) with probes specific for fusion gene breakpoints, unarranged alleles, or IGH translocation breakpoints. The *EML4-ALK* fusion was clonal being present in ~50% of the DNA molecules, equivalent to one allele having the rearrangement. *ARHGAP27-MAP3K14* fusion was subclonal (10% of DNA, 20% of cells), as were *KANK1-BRAF* (15%/30%; 16%/32% after adjusting for purity) and *GTF2I-BRAF* (13%/26%; 19%/38% after adjusting for purity)

We went on to determine if the kinase fusion genes were also subclonal by performing droplet digital PCR using breakpoint-specific sequences. DNA level breakpoints were available for four samples with kinase fusions and were tested for both the rearranged and wild-type alleles (Fig. 4). Taking into account the copy number of the involved chromosomes, the *EML4-ALK* fusion was fully clonal, being present in 55% of the droplets. This patient did not have any other identified MEK/ERK abnormalities but did have a t(14;16) suggesting that the *EML4-ALK* fusion was not an initiating event and had been selected for during progression. This patient was GEP70 high risk, but did not have gain of 1q or deletion of *TP53*, however there was a subclonal mutation in *TP53* (p.S240R, 2% of reads). The three other patients all had subclonal kinase fusion genes (range 20–38% of cells) and had subclonal mutations in the Ras/Raf pathway genes, indicating parallel evolution of MEK/ERK pathway activation in these patients. One patient had three samples sequenced over a 5-month period and showed consistent expression of the *TSPAN3-ROS1* fusion gene in all samples.

### Overexpression of *NIK* through an *IGH* translocation

Fusion genes involving NIK were found in this dataset and in the CoMMpass dataset. We also noticed translocations involving *NIK* in 2/98 (2%) samples from another targeted panel dataset, indicating multiple mechanisms of kinase activation. The translocations were t(14;17)(q32.33;q21.31) involving the *IGH* locus and resulted in overexpression of *NIK*. Both samples with the t(14;17) were hyperdiploid and did not have an additional *IGH* translocation. Based on a larger dataset of 414 samples, we saw spiked expression of *NIK* in nine samples, indicating that the frequency of increased kinase expression through the t(14;17) is 2.2% (Fig. 3a). Of these nine samples, there were four hyperdiploid samples, two t(4;14), and one of each t(11;14), t(14;16), and t(14;20). A similar spike in NIK expression was seen in the CoMMpass RNA-sequencing data (*n* = 734 NDMM) in 3.2% of patients, which coincided with both Ig and non-Ig translocations in those samples (Fig. 3b). The prevalence of primary *IGH* translocations alongside the t(14;17) indicates that the *NIK* translocations are secondary events (Table 2).

## Discussion

We show for the first time that, analogous to other cancers, kinase domain fusion genes are seen in MM occurring at a similar frequency, ~1.5% of cases. Many of the fusion genes described here have been shown in other cancers to affect the MEK/ERK, PIK3CA, and NF-κB pathways, and are therefore likely to be functional in MM too.

Each of the pathways described is of relevance in MM. Here, we found fusion genes involving *BRAF*, *NTRK3*, *ALK*, *FGFR1*, and *ROS1*, which result in activation of the MEK/ERK pathway in other cancers [20]. Even though the kinase domain-containing genes are diverse, they all have similar domain structures and feed into the same downstream pathway that makes them targetable. This is analogous to adenocarcinoma of the lung where fusion genes involving MET, ROS1, and ALK are all treated with TKIs that are effective against each of the kinase domains.

The MEK/ERK pathway is commonly disrupted in MM, with activating point mutations seen in *KRAS*, *NRAS*, and *BRAF* in ~50% of patients [13, 21]. In samples with kinase fusion genes, we only identified subclonal mutations in *KRAS*, *NRAS*, and *BRAF*, consistent with the hypothesis that these mechanisms are mutually exclusive and serve the same functional purpose, constituting a parallel mechanism for pathway activation.

It is highly likely that the fusion genes described here are secondary events, due to their subclonal nature or association with primary translocations. However, these fusions were detected in SMM, as well as NDMM and relapse patients, indicating that they are not necessarily late progression events. A *ROS1* fusion gene was detected in a SMM patient. *ROS1* is a RTK that activates the MEK/ERK pathway through phosphorylation of Ras. *ROS1* fusions are detected in ~2% of non-small cell lung carcinoma (NSCLC) patients [20]. Another key RTK in NSCLC is *ALK*, which was also identified in this dataset as a fusion gene. These RTK fusion genes are considered as driver events in NSCLC, as they are prevalent, activating, and often clonal in nature [22]. The *EML4-ALK* fusion gene was clonal in our MM sample, indicating that it is also a driver event in MM and may be a relevant clinical target in a small subset of patients.

*NTRK1* gene expression was increased by gene fusion events, and we also saw that in total 0.5% of patient samples in the CoMMpass dataset had increased expression of *NTRK1*. It is unclear if this increase in expression is solely due to gene fusions with *NTRK1* or if there are other genetic or epigenetic events contributing to this. However, this small subset of patients with this abnormality may benefit from TKI treatment options.

Both *ROS1, NTRK1*, and *ALK* fusion genes are treated using TKIs, such as crizotinib, in NSCLC with varying degrees of success, due to genetic heterogeneity. Many patients with *ALK* fusions in NSCLC respond to TKI treatment, but later relapse [23], due to succession of a clone with a mutation in the ALK tyrosine kinase domain or a clone with an alternative MEK/ERK signaling abnormality [24]. Genetic heterogeneity in MM is well described [25–27] and likely poses a similar problem in the treatment of patients with these secondary, subclonal events.

There was also evidence of NF-κB activation through fusion genes and Ig translocations involving NIK, which is a central component of the non-canonical NF-κB pathway [28]. NIK is normally targeted for degradation by the proteasome through the TRAF-cIAP destruction complex, and accumulation of NIK is associated with lymphoid malignancies [29]. It has been shown that negative regulators of the non-canonical pathway are frequently deleted or mutated in MM, including members of the TRAF-cIAP complex: TRAF2, TRAF3, BIRC2 (cIAP1), and BIRC3 (cIAP2) [13, 30, 31]. Activation of NIK in myeloma cell lines and patients has been seen through inactivation of this complex, but NIK translocations have only been noted in cell lines [30, 32]. We see here two means of NIK degregulation: firstly through overexpression with the t(14;17) Ig translocation, and secondly with fusion genes that result in loss of the BIRC2 binding domain that regulates NIK degradation. Taken together, these data indicate that NIK is a key player in regulating the non-canonical NF-κB pathway. Both the NIK fusion gene and the translocations are secondary events as they are either subclonal or the sample has a primary Ig translocation, indicating that this could be a key progression event.

The fusion genes described here were detected using a panel targeted against therapeutically tractable fusions that have been previously identified in other cancers. The probability of generating an in-frame fusion gene is 33%, therefore of the 39 fusion genes detected in this study, 13 may be in-frame by chance, and so out of the 21 in-frame fusions detected, 8 may be biologically relevant. However, as these were detected using a targeted panel using prior information from other cancer types, we have enriched for biologically relevant genes and we believe that all of the kinase fusions are biologically relevant. Indeed, fusion genes mostly involving Ig loci have recently been described and are associated with outcome, although no functional fusion genes were described [12]. These may be markers for genome instability rather than have any new biological function from the gene fusions.
